# Weed infestation and productivity of wheat crop sown in various cropping systems under conventional and conservation tillage

**DOI:** 10.3389/fpls.2023.1176738

**Published:** 2023-07-13

**Authors:** Waqas Ahmed Minhas, Naima Mumtaz, Hafeez Ur-Rehman, Shahid Farooq, Muhammad Farooq, Hayssam M. Ali, Mubshar Hussain

**Affiliations:** ^1^ Department of Agronomy, Bahauddin Zakariya University, Multan, Pakistan; ^2^ Department of Agronomy, University of Agriculture, Faisalabad, Pakistan; ^3^ Department of Plant Protection, Faculty of Agriculture, Harran University, Şanlıurfa, Türkiye; ^4^ Department of Plant Sciences, College of Agricultural and Marine Sciences, Sultan Qaboos University, Al Khoud, Oman; ^5^ Department of Botany and Microbiology, College of Science, King Saud University, Riyadh, Saudi Arabia; ^6^ School of Veterinary and Life Sciences, Murdoch University, Murdoch, WA, Australia

**Keywords:** wheat, tillage, cropping system, soil properties, grain yield, grain quality

## Abstract

**Introduction:**

Climate change, pest infestation, and soil degradation are significantly reducing wheat (*Triticum aestivum* L.) yield. Wheat is cultivated in rice-wheat and cotton-wheat cropping systems and escalating global population is exerting substantial pressure on the efficiency of these systems. Conservation tillage and crop rotation could help in lowering soil degradation and pest infestation, and improving wheat yield.

**Methods:**

This three-year study evaluated soil properties, weed infestation and wheat yield under various tillage and cropping systems. Six different cropping systems, i.e., cotton-wheat, sorghum-wheat, mungbean-wheat, rice-wheat, sunflower-wheat, and fallow-wheat (control) and three tillage systems, i.e., conventional tillage (CT), zero-tillage (ZT) and minimum tillage (MT) were included in the study.

**Results:**

The individual and interactive effects of tillage and cropping systems significantly affected soil properties, weed infestation and yield of wheat crop. Overall, CT resulted in lower soil bulk density and higher porosity, while ZT behaved oppositely at all locations in this regard. Similarly, mungbean-wheat cropping system resulted in lower bulk density and higher porosity and nitrogen (N) contents, while fallow-wheat cropping system resulted in higher bulk density, and lower soil porosity and N contents. Similarly, ZT and CT resulted in higher and lower weed infestation, respectively. Likewise, lower and higher weed density and biomass were recorded in wheat-sorghum and wheat-fallow cropping systems, respectively at all locations. In the same way higher number of productive tillers, number of grains per spike, 1000-grain weight, grain yield, and economic returns of wheat crop were recorded for CT, whereas ZT resulted in lower values of these traits. Regarding interactions, wheat-mungbean cropping system with CT resulted in lower bulk density and higher porosity and N contents, whereas wheat-fallow system with ZT behaved oppositely at all locations in this regard. Similarly, higher and lower values for yield-related traits and economic returns of wheat crop were noted for mungbean-wheat cropping system under CT and fallow-wheat and sorghum-wheat cropping systems under ZT, respectively. It is concluded that the mungbean-wheat cropping system improved wheat productivity and soil health and sorghum-wheat cropping system could lower weed infestation. Therefore, these cropping systems can be practiced to lower weed infestation and improve wheat yield and economic returns.

## 1 Introduction

Pakistan has 6^th^ largest human population in the world and wheat (*Triticum aestivum* L.) is consumed as staple diet in the country. Wheat is cultivated on 9.78 million ha in Pakistan with a gross domestic product (GDP) share of 1.8%. However, 2.1% decrease in the area dedicated to wheat cultivation and 3.9% decrease in annual production was recorded during 2021-22 in the country (GOP, 2021). The global population is projected to increase by 2.4 billion individuals by the year 2050, indicating a significant growth rate (Smith, 2015). Moreover, wheat production is stagnant in many countries across the globe (Brisson et al., 2010), including Pakistan which is alarming. Two major cropping systems, i.e., rice (*Oryza sativa* L.)-wheat and cotton (*Gossypium hirsutum* L.)-wheat are being practiced in Pakistan for several decades. Wheat cultivation is a significant component of these cropping systems, and the escalating global population is exerting substantial pressure on the efficiency of these systems. Indeed, there is delay in wheat sowing due to late maturity and harvesting of kharif crops (cotton and rice) in both cropping systems, which results in significant yield reduction of wheat crop (Bismillah Khan et al., 2010; Hussain et al., 2012a; Hussain et al., 2012b). Moreover, there is an edaphic conflict in rice-wheat cropping system as rice needs well-puddled soil, while the wheat crop requires well-drained pulverized soil (Nawaz et al., 2019). The burning of previous crop residues is another serious problem in these cropping systems which causes greenhouse gases’ emission resulting in serious threats to environment (Mandal et al., 2004). Furthermore, crops cultivated in these cropping systems are exhaustive and deplete soil nutrient reserves. Moreover, these cropping systems have been following conventional tillage (CT) practices for several decades which have enhanced weed infestation (Shahzad et al., 2016a; Shahzad et al., 2016b; Nawaz et al., 2019).

In this scenario, introduction of new crops or alternative cropping systems and soil management practices capable of minimizing the delay in sowing of wheat crop, proper management of crop resides, improvement in soil health, and effective weed management are direly needed. Conservation agriculture practices in rice-wheat and cotton-wheat cropping systems could serve as an alternative to conventional agronomic management (conventional tillage in particular) (Shahzad et al., 2016a; Nawaz et al., 2019). Moreover, modification in the cropping systems may help in reducing yield losses. Incorporation is the suitable way to discard the residues of previous crop and save time (Jiang et al., 2001). Residue incorporation could enhance soil fertility and productivity, ultimately resulting in improved crop yields (Wang et al., 2001). Therefore, a cropping system capable of improving soil health and wheat yield and lowering weed infestation is needed. Crop diversification and novel cropping systems can lower weed infestation, improve soil health and overall system productivity (Naeem et al., 2021a; Naeem et al., 2021c). Several studies have reported that adding a leguminous crop in the cropping system improved yield of the following crops (Naeem et al., 2021a; Naeem et al., 2021b; Naeem et al., 2021c). Similarly, adding a crop with allelopathic effects improved weed management (Shahzad et al., 2016a).

Tillage practices can alter soil organic matter content, soil nutrient supply and soil microbial activity (Abdalla et al., 2013). Conventional tillage (CT) is more effective in enhancing soil fertility status; however, badly affects soil structure, create hardpan, cause soil compactions and erosion if practiced for extended periods (Haruna and Nkongolo, 2020). Continuous use of CT for crop production results in the development of a hard sub-surface layer, which results in reduced yields due to the low moisture and nutrient uptake by crop plants (Bertolino et al., 2010). The CT helps in weed eradication (Gajri et al., 1999) during the initial crop growth stages; however, weed infestation becomes a serious problem at later crop growth stages. The conservation agriculture practices including minimum tillage (MT) and zero tillage (ZT) reduce production costs by lowering the costs incurred on land preparation, fuel, labor, and farm equipment. Moreover, CT ensures water and soil conservation (Jabran and Aulakh, 2015). Agronomic practices had a great impact in the success of conservation agriculture (Oyeogbe et al., 2017). The ZT involves direct sowing of seeds into untilled soil, as opposed to traditional methods such as plowing or conventional seedbed preparation. The ZT is regarded as a sustainable tillage practice that provides several environmental and agronomic advantages. Reduced soil disturbance has numerous benefits for sustainable farming systems, including supporting soil health, conserving water, sequestering carbon, and promoting biodiversity. The ZT seems a feasible option which could reduce cultivation cost and avoid 2-3 weeks delay in wheat through direct planting without soil preparation (Sidhu et al., 2007). Stošić et al. (2021) reported that reduced or ZT can be good alternative to CT because it provides balance between greenhouse gas emissions, crop yields and nitrogen balances.

Weed infestation is a major cause of yield reduction in wheat crop (Jiang et al., 2001; Shahzad et al., 2016b). Indeed, weeds compete with crop plants, reduce yields and deteriorate grain quality (Verma et al., 2015). According to Shahzad et al. (2016b), fallow-wheat cropping system favored grassy and broad-leaved weed species, whereas some other cropping systems favor the proliferation of specific weed species. Therefore, primary principle of sustainable weed management is to change or break the life cycle to reduce weed infestation and weed-crop competition (Shahzad et al., 2016a; Shahzad et al., 2016b). Crop rotation, use of allelopathic crops and mulching are some of the agronomic practices capable of lowering weed infestation. These practices are effective in reducing weed growth and reproduction leading to lower weed infestation (Jabran, 2017a). Crop rotation helps to interrupt the weeds and main crop relationship and breaks the weed cycle. Sorghum, sunflower, mulberry and eucalyptus are well known allelopathic crops and proved effective in weed control (Shahzad et al., 2016b; Jabran, 2017a). Allelochemicals are once released in soil or plant it badly effects the target plant abilities (Kruse et al., 2000). Tillage is one of the key elements affecting weed infestation in various crops (Sekutowski and Smagacz, 2014). Tillage is known to induce the germination of weed seeds. Additionally, distinct tillage systems have been found to have varying impacts on the aggregation and dispersion of weed seeds across diverse soil strata (Benech-Arnold et al., 2000; Ghersa and Martınez-Ghersa, 2000). Conventional tillage results in the uniform dispersion of weed seeds throughout the plow layer, whereas reduced tillage tend to concentrate the seeds in the uppermost soil layer. The concentration of weeds seeds in the uppermost layer facilitates their germination because of high weed infestation is observed in initial years of reduced tillage (Benech-Arnold et al., 2000). Continuous reduced or no tillage depletes seed bank in the upper soil layer; thus, results in lower weed infestation in the later years.

Although the individual effects of different alternative cropping systems and tillage practices have been explored on weed flora in wheat crop and soil health, no comprehensive study investigated weed infestation and productivity of these cropping systems under various tillage practices. Therefore, this study recorded weed infestation in wheat crop, yield-related traits of wheat crop, and soil health under various alternative cropping systems and tillage practices. It was hypothesized that cropping system having leguminous crop combined with CT will improve soil health and wheat yield, whereas the cropping system containing crops with allelopathic potential combined with ZT would lower weed infestation. The results will help to select the cropping systems capable of improving wheat yield and soil health and lowering weed infestation.

## 2 Materials and methods

This study was conducted out at three different experimental sites located in three distinct agroclimatic zones of Pakistan. The Multan site was at Department of Agronomy Farm, Bahauddin Zakariya University Multan. The Faisalabad site was located at Research Farm, University of Agriculture Faisalabad. Similarly, Hafizabad site was located at a farmer field in district Hafizabad. The experiment was conducted during 2019-20, 2020-21 and 2021-22. The soil properties of the experimental sites are given in **Table 1**. The soils of the experimental sites had no slopes as these had been precision levelled prior to the initiation of current study.

**Table 1:** Soil properties of different experimental sites included in the study.

Soil properties	Experimental sites		
	Multan	Faisalabad	Hafizabad
Soil texture	Loamy	Sandy-clay-loam	Sandy-loam
pH	8.3	7.9	8.0
EC (mS cm^−1)^	2.81	1.35	3.20
Organic matter (%)	0.81	0.76	0.67
Total nitrogen (mg kg^-1^)	0.103	0.05	0.049
Available phosphorus (mg kg^-1^)	7.85	6.74	6.78
Available potassium (mg kg^-1^)	200	181	175

### 2.1 Experiment details

Six different cropping systems, i.e., cotton-wheat, sorghum-wheat, mungbean-wheat, rice-wheat, sunflower-wheat, and fallow-wheat (control) were included in the study. Similarly, three different tillage systems, i.e., conventional tillage (CT), zero-tillage (ZT) and minimum tillage (MT) were used to grow both winter and summer crops in these cropping systems. The principles of conservation tillage, i.e., reduction in tillage intensity, soil cover and crop rotation were followed. Crop residues were left as such in ZT and MT treatments and tillage intensity was reduced in both these treatments compared to CT. Similarly, different kharif (summer) crops were included in the rotation. All kharif crops (**Table 2**) included in the opted cropping systems were sown at their recommended sowing time and harvested at harvest maturity. Wheat was successively sown after all kharif crops and fallow period. In conventional tillage (CT), the seedbed was prepared by cultivating the field twice with a tractor-mounted plough, followed by planking. Wheat seeds were sown using a tractor-drawn happy seeder machine or a zero-drill machine under minimum tillage (MT) and zero tillage (ZT), respectively. The experiment had three replications with a net plot size of 5.0 m × 2.7 m. The experiment was laid out using a randomized complete block design (RCBD) with a spilt plot design. Tillage systems were maintained in the main plots, whereas the cropping systems were randomly assigned to sub-plots.

**Table 2:** Agronomic practices used for different kharif crops (summer) and wheat (winter crop) included in the current study.

Sowing time	Crops					
	Wheat	Cotton	Mungbean	Sorghum	Rice	Sunflower
2019-20	23, 26, 28 Nov*	22 May	15 June	22 May	9 July	24 July
2020-21	26, 29, 31 Nov	19 May	14 June	23 May	10 July	22 July
2021-22	24, 27, 29 Nov	17 May	16 June	25 May	12 July	20 July
NPK (kg ha^-1^)	150-100-80	200-150-100	30-60	100-60	140-80-65	150-60-60
P-P spacing (cm)	–	15	10	20	–	25
R-R spacing (cm)	22.5	75	30	45	25	75
Seed rate (kg ha^-1^)	150	25	20	20	80	6
TSW 2019-20	44.12	77.81	57.12	23.45	19.91	41.23
TSW 2020-21	45.21	78.34	58.21	22.61	20.04	40.15
TSW 2021-22	43.65	77.12	57.56	24.33	20.12	41.63
G % 2019-20	98	92	96	90	97	98
G % 2020-21	98	91	97	90	97	98
G % 2021-22	98	92	96	90	97	98

*Different dates indicate sowing times for Multan, Hafizabad and Faisalabad experimental sites, respectively, P-P spacing, plant-to-plant spacing, R-R spacing, row-to-row spacing, - in P-P spacing row indicate that there was no P-P spacing for the respective crop, TSW, 1000-seed weight, G %, seed germination percentage as cliamed by the seed providers.

### 2.2 Crop husbandry

Kharif crops were sown on well-prepared seedbeds following the recommended agronomic practices, while no crop was sown during fallow period. Before sowing of each kharif crop, pre-soaking irrigation was applied to the field. The seedbed was prepared (for CT) according to the tillage systems (or no seedbed preparation in case of MT and ZT) after soil reached workable moisture regime and kharif crops were sown. Crops were irrigated according to their moisture requirements. All kharif crops were harvested at their harvest maturity.

Wheat crop was sown either on well-prepared seedbed for CT, whereas happy seeder machine or a zero-drill machine were used for the sowing of wheat crop in MT and ZT, respectively. Seed rate, sowing time, fertilizers, row-to-row, and plant-to-plant spacing used for wheat crop are given in **Table 2**. Three separate splits of nitrogen (N) were applied at the time of seeding, and with the first and second irrigations. The whole amounts of phosphorus (P) and potassium (K) was supplied during wheat planting. Wheat received five irrigations. The recommended cultural and agronomic measure was taken to safeguard wheat crop against pests and diseases.

### 2.3 Soil physical properties

Following wheat harvest, soil samples were taken using a soil core sampler to determine soil bulk density (BD) and total porosity. Three random samples were collected from 0 to 15 cm soil depth in each experimental plot. The collected samples were mixed and dried for 24 hours at 105°C. Blake and Hartge (1986) were followed to measure the BD. Soil porosity was calculated according to the methods described by Danielson and Sutherland (1986).

### 2.4 Nutrient availability

Three random soil samples were collected from a depth of 15 cm in each sub-plot following wheat harvest during each year. The collected samples were mixed to get a composite sample for analyzing total available N in the soil. Soil nitrate nitrogen (NO_3_-N) and ammoniacal nitrogen (NH_4_-N) were determined by using standard AB-DTPA (Ammonium Bicarbonate-DTPA) method devised by Soltanpour and Schwab (1977) and modified by Soltanpour and Workman (1979).

### 2.5 Weed infestation

Weed infestation was assessed in terms of density and biomass of the recorded weed species in wheat crop. Weed infestation data were recorded at two different times, i.e., 45 and 65 days after sowing (DAS). Three locations were randomly selected from each experimental unit using a 1 m × 1 m quadrat. All weed plants that settled in the quadrat were collected and counted to determine density. Afterwards, the collected plants were dried in an oven at 70 ± 5°C until constant weight (Shahzad et al., 2016b).

### 2.6 Agronomic and yield-related traits of wheat

The quadrat method was used to record the density of productive tillers. Spike-bearing tillers were counted by randomly placing 1 m^2^ quadrat in each experimental unit at three different places and averaged. Twenty-five randomly chosen spikes from each experimental unit were used to determine the average number of grains per spike. Additionally, the weights of 1000 grains were measured for 5 randomly selected samples from each experimental unit. A total 5 1000 grain samples from each experimental unit were weighed, and their weight was averaged. Biological yield was determined by harvesting each experimental unit, letting it dry in the sun for two days, and then weighing it using a spring scale. Grain yield was calculated by weighing the grains that were manually threshed from the sun-dried samples. Grain yield was calculated in tons per hectare using the unitary method (Farooq et al., 2015).

### 2.7 Statistical analysis

The collected data on soil properties, weed density and biomass and yield-related traits were tested for normality and homogeneity of variance (Shapiro and Wilk, 1965). The data were normally distributed; therefore, original data were used in statistical analysis. The data of different locations were analyzed separately since they had different soil properties and located in different agro-ecological zones. The year effect was non-significant; therefore, data of different years were pooled for the analysis. Two-way analysis of variance (ANOVA) (Steel et al., 1997) was used to analyze the data of each location, separately. The least significant difference (LSD) at the 95% confidence level was used to compare the means of the individual and combined impacts of cropping and tillage methods where ANOVA showed significant differences. All statistical analyses were conducted on SPSS statistical software version 21.0.

## 3 Results

The individual and interactive effects of Tillage (T) and cropping systems (C) significantly altered soil bulk density (BD), soil porosity (PO), and total available nitrogen (N) at all locations included in the current study (**Tables 3**, **4**). Zero tillage (ZT) resulted in the highest BD, whereas the lowest BD was recorded for conventional tillage (CT) at all locations. Similarly, the highest and the lowest BD was recorded for fallow-wheat and mungbean-wheat cropping system, respectively at all locations. Regarding T × C interaction, fallow-wheat cropping system with ZT resulted in the highest BD, whereas mungbean-wheat cropping system with CT resulted in the lowest values of BD at all locations. In contrast to BD, CT and mungbean-wheat cropping system resulted in the highest PO, while ZT and fallow-wheat cropping system resulted in the lowest values of PO at all locations. Similarly, CT × mungbean-wheat cropping system recorded the highest values of PO at all locations, whereas ZT × fallow-wheat cropping system resulted in the lowest PO at all locations (**Table 3**). ZT and CT resulted in the highest and the lowest total N, respectively at all locations. Similarly, the highest and the lowest total available N was recorded for mungbean-wheat and fallow-wheat cropping systems, respectively at all locations. The T × C interaction indicated that mungbean-wheat cropping system under ZT recorded the highest, while fallow-wheat cropping system with CT recorded the lowest total soil available N at all locations (**Table 4**).

**Table 3:** Individual and interactive effects of different tillage and cropping systems on soil bulk density and soil porosity after the harvest of wheat crop at different locations included in the study.

Cropping systems	CT	ZT	MT	Means (CS)	CT	ZT	MT	Means (CS)
	Bulk density (g cm^−3^)				Porosity (%)			
MULTAN								
Cotton-W	1.41 ij	1.49 b	1.46 fg	1.45 C	41.01 cd	37.46 j	39.14 g	39.20 C
Sorghum-W	1.41 ij	1.48 bc	1.46 f	1.45 C	40.81 cd	37.43 j	39.03 g	39.09 C
Mungbean-W	1.36 k	1.46 f	1.43 h	1.42 D	44.45 a	39.62 f	40.63 d	41.57 A
Rice-W	1.41 i	1.49 b	1.48 de	1.46 B	41.19 bc	37.74 ij	38.89 g	39.28 C
Sunflower-W	1.40 j	1.48 cd	1.47 e	1.45 C	41.46 b	38.08 hi	39.23 fg	39.59 B
Fallow-W	1.44 g	1.51 a	1.47 e	1.48 A	40.09 e	36.21 k	38.42 h	38.24 D
Means (TS)	1.41 C	1.49 A	1.46 B		41.50 A	37.76 C	39.22 B	
LSD at 5% for TS = 0.02; CS = 0.02; TS × CS = 0.01					LSD at 5% for TS = 0.19; CS = 0.22; TS × CS = 0.38			
HAFIZABAD								
Cotton-W	1.45 j	1.54 b	1.50 fg	1.49 C	33.51 cd	30.61 j	31.99 g	31.94 C
Sorghum-W	1.45 ij	1.53 bc	1.51 f	1.49 C	33.35 d	30.59 j	31.89 g	31.94 C
Mungbean-W	1.41 k	1.51 f	1.48 h	1.46 D	36.33 a	32.38 f	33.20 d	33.97 A
Rice-W	1.46 i	1.54 b	1.52 c-e	1.51 B	33.66 bc	30.85 ij	31.79 g	32.10 C
Sunflower-W	1.45 j	1.53 cd	1.52 e	1.50 C	33.88 b	31.12 ji	32.06 fg	32.35 B
Fallow-W	1.49 g	1.55 a	1.52 de	1.52 A	32.76 e	29.59 k	31.40 h	31.25 D
Means (TS)	1.45 C	1.53 A	1.51B		33.91 A	30.86 C	32.05 B	
LSD at 5% for TS = 0.02; CS = 0.01; TS × CS = 0.01					LSD at 5% for TS = 0.16; CS = 018; TS × CS = 0.31			
FAISALABAD								
Cotton-W	1.45 ij	1.54 b	1.50 fg	1.50 C	39.93 c	36.07 e	37.34 d	37.78 CD
Sorghum-W	1.45 ij	1.53 b	1.51 f	1.50 C	39.77 c	36.09 e	37.44 d	37.77 D
Mungbean-W	1.41 k	1.51 f	1.50 g	1.47 D	42.31 a	39.49 c	39.49 c	39.91 A
Rice-W	1.46 i	1.54 b	1.52 e	1.51 B	39.97 c	36.29 e	37.84 d	38.03 CD
Sunflower-W	1.45 j	1.53 cd	1.52 e	1.50 C	40.93 b	36.03 e	37.70 d	38.22 BC
Fallow-W	1.50 fg	1.56 a	1.52 de	1.53 A	40.80 b	37.16 d	37.82 d	38.60 B
Means (TS)	1.45 C	1.53 A	1.51 B		40.62 A	36.59 C	40.61 B	
LSD at 5% for TS = 0.02; CS = 0.01; TS × CS = 0.01					LSD at 5% for TS = 0.47; CS = 45; TS × CS = 0.78			

Here, CT, conventional tillage; ZT, zero-tillage; MT, minimum tillage; CS, cropping systems; TS, tillage systems; W, wheat. Means followed by different letters are statistically different from each other within each location. The data of locations were analyzed separately due to differences in soil, climate, and agroecology. The values presented are means of three-year data.

**Table 4:** Individual and interactive effects of different tillage and cropping systems on total available nitrogen in soil after the harvest of wheat crop and number of productive tillers of wheat crop sown at different locaitons included in the study.

Cropping systems	CT	ZT	MT	Means (CS)	CT	ZT	MT	Means (CS)
	Total nitrogen (mg kg^-1^)				Productive tillers/m^2^			
MULTAN								
Cotton-W	8.37 o	10.28 d	9.62 h	9.42 D	155.17 e-h	128.12 kl	141.90 h-k	141.73 CD
Sorghum-W	8.53 n	10.09 e	9.54 i	9.39 D	161.50 d-g	133.58 i-l	151.83 f-h	148.97 C
Mungbean-W	9.39 j	11.05 a	10.22 d	10.22 A	226.48 a	167.32 d-f	175.77 cd	189.86 A
Rice-W	8.62 m	10.43 c	9.80 g	9.62 C	185.70 bc	149.07 g-i	170.37 c-e	168.21 B
Sunflower-W	9.03 l	10.53 b	9.96 f	9.84 B	197.53 b	144.78 h-j	162.32 d-g	168.21 B
Fallow-W	8.07 p	9.93 f	9.28 k	9.09 E	148.07 g-j	120.53 l	133.03 j-l	133.88 D
Means (TS)	8.67 C	10.39 A	9.74 B		179.08 A	140.57 C	155.87 B	
LSD at 5% for TS = 0.04; CS = 0.05; TS × CS = 0.08					LSD at 5% for TS = 7.24; CS = 8.97; TS × CS = 15.83			
HAFIZABAD								
Cotton-W	6.57 l	8.09 c	7.57 f	7.41 D	218.33 c	178.20 g	187.50 ef	194.68 C
Sorghum-W	6.67 k	7.92 d	7.49 g	7.36 E	201.63 d	147.30 i	168.73 gh	172.63 D
Mungbean-W	7.40 g	8.67 a	8.08 c	8.05 A	254.30 a	196.03 de	215.43 c	221.92 A
Rice-W	6.80 j	8.14 c	7.71 e	7.54 C	231.97 b	185.87 f	200.50 d	206.11 B
Sunflower-W	7.12 i	8.29 b	7.84 d	7.75 B	219.90	173.83 gh	193.73 d-f	195.82 C
Fallow-W	6.37 m	7.73 e	7.26 h	7.12 F	196.17 de	135.33 j	169.73 gh	16.08 E
Means (TS)	6.82 C	8.14 A	7.66 B		220.38 A	169.43 C	189.31 B	
LSD at 5% for TS = 0.08; CS = 0.04; TS × CS = 0.103					LSD at 5% for TS = 7.05; CS = 3.85; TS × CS = 9.21			
FAISALABAD								
Cotton-W	8.88 o	10.96 d	10.26 h	10.03 D	257.67 c	200.67 f	222.50 e	226.94 C
Sorghum-W	9.12 n	10.74 e	10.16 i	10.01 D	238.67 d	178.83 h	204.00 f	207.17 D
Mungbean-W	10.02 j	11.80 a	10.90 d	10.90 A	292.00 a	219.20 e	246.67 d	252.72 A
Rice-W	9.20 m	11.12 c	10.45 g	10.25 C	266.50 b	203.17 f	226.83 e	232.22 B
Sunflower-W	9.64 l	11.22 b	10.62 f	10.49 B	265.50 b	200.50 f	221.67 e	229.22 BC
Fallow-W	8.61 p	10.57 f	9.89 k	9.69 E	226.83 e	154.50 i	190.33 g	190.50 E
Means (TS)	9.24 C	11.07 A	10.38 B		257.86 A	192.86 B	192.86 C	
LSD at 5% for TS = 0.05; CS = 0.05; TS × CS = 0.09					LSD at 5% for TS = 6.24; CS = 4.14; TS × CS = 8.94			

Here, CT, conventional tillage; ZT, zero-tillage; MT, minimum tillage; CS, cropping systems; TS, tillage systems; W, wheat. Means followed by different letters are statistically different from each other within each location. The data of locations were analyzed separately due to differences in soil, climate and agroecology. The values presented are means of three-year data.

Individual and interactive effects of T and C substantially influenced yield-related characteristics and grain protein contents of wheat crop at all locations except for the non-significant T × C interaction for grain protein contents at all sites, and number of grains per spike in Multan (**Tables 4**–**6**).

**Table 5:** Individual and interactive effects of different tillage and cropping systems on number of grains per spike and 1000- grain weight of wheat crop at different locations included in the study.

Cropping systems	CT	ZT	MT	Means (CS)	CT	ZT	MT	Means (CS)
	Number of grains per spike				1000-grain weight (g)			
MULTAN								
Cotton-W	49.17	41.92	46.36	45.82 C	39.93 d-f	38.25 hi	39.26 fg	39.15 BC
Sorghum-W	46.17	41.22	45.10	44.17 D	38.70 gh	37.79 hi	39.50 e-g	38.67 DE
Mungbean-W	52.57	46.84	49.97	49.79 A	43.80 a	40.38 c-e	41.65 b	41.94 A
Rice-W	49.97	43.24	47.69	46.96 B	41.33 bc	38.14 hi	40.17 d-f	39.88 B
Sunflower-W	49.24	43.39	47.69	46.77 B	40.72 b-d	38.25 hi	39.26 fg	39.41 BC
Fallow-W	46.44	41.13	44.62	44.06 D	38.11 hi	37.39 i	39.88 d-f	38.46 E
Means (TS)	48.93 A	42.6 C	46.90 B		40.43 A	38.37 B	39.95 C	
LSD at 5% for TS = 1.26; CS = 0.90; TS × CS = NS					LSD at 5% for TS = 0.34; CS = 0.58; TS × CS = 0.98			
HAFIZABAD								
Cotton-W	49.74 b	44.51 d-f	47.08 c	46.96 B	39.88 b-d	37.27 gh	38.91 e	38.92 B
Sorghum-W	44.93 d	40.11 g	44.51 de	43.18 C	38.07 f	36.54 h	37.85 fg	37.49 D
Mungbean-W	54.49 a	46.88 c	49.70 b	50.35 A	43.12 a	39.18 de	40.55 b	40.95 A
Rice-W	50.58 b	43.29 f	46.93 c	46.93 B	40.43 bc	36.97 h	39.40 de	38.92 B
Sunflower-W	49.58 b	44.09 d-f	46.75 c	45.81 B	39.78 cd	37.05 h	38.06 f	38.30 C
Fallow-W	44.60 de	39.97 g	43.77 ef	42.78 C	38.09 f	36.58 h	36.97 h	37.21 D
Means (TS)	48.99 A	43.07 C	46.46 B		39.89 A	37.26 C	38.62 B	
LSD at 5% for TS = 0.27; CS = 0.61; TS × CS = 0.10					LSD at 5% for TS = 0.23; CS = 0.45; TS × CS = 0.75			
FAISALABAD								
Cotton-W	48.42 ef	45.05 ij	47.28 fg	46.92 C	40.21 d	37.89 g	38.90 e	39.00 C
Sorghum-W	46.79 gh	44.23 j	47.48 fg	46.17 D	38.91 e	37.43 gh	38.69 ef	38.34 D
Mungbean-W	55.68 a	50.36 c	52.53 b	52.86 A	43.62 a	40.02 d	41.14 bc	41.59 A
Rice-W	50.27 c	47.35 fg	49.52 c-e	49.04 B	41,79 b	37.78 gh	40.24 d	39.93 B
Sunflower-W	49.76 cd	47.26 f-h	48.93 de	48.65 B	40.62 cd	37.89 g	38.90 e	39.14 C
Fallow-W	44.87 ij	42.72 k	46.08 hi	44.56 E	38.12 fg	37.03 h	38.01 fg	37.72 E
Means (TS)	49.30 A	46.16 C	48.64 B		40.54 A	38.01 C	39.31 B	
LSD at 5% for TS = 0.66; CS = 0.68; TS × CS = 1.19					LSD at 5% for TS = 0.27; CS = 0.44; TS × CS = 0.75			

Here, CT, conventional tillage; ZT, zero-tillage; MT, minimum tillage; CS, cropping systems; TS, tillage systems; W, wheat, NS, non-significant. Means followed by different letters are statistically different from each other within each location. The data of locations were analyzed separately due to differences in soil, climate and agroecology. The values presented are means of three-year data.

**Table 6:** Individual and interactive effecrs of different tillage and cropping systems on grain yield and protein content after wheat crop sown at different locations included in the current study.

Cropping systems	CT	ZT	MT	Means (CS)	CT	ZT	MT	Means (CS)
	Grain yield (t ha^-1^)				Grain protein content (%)			
MULTAN								
Cotton-W	4.65 c	3.76 h	4.08 ef	4.16 C	22.89	17.19	21.43	20.50 C
Sorghum-W	4.02 fg	3.00 j	3.37 i	3.46 D	21.43	16.69	18.63	18.46 D
Mungbean-W	5.54 a	4.17 ef	4.41d	4.70 A	26.80	24.12	24.22	25.04 A
Rice-W	4.78 bc	3.50 i	4.24 de	4.17 C	23.99	19.36	22.21	21.85 B
Sunflower-W	4.95 b	3.86 gh	4.12 ef	4.31 B	22.65	17.95	20.41	20.34 C
Fallow-W	4.13 ef	3.03 j	3.49 i	3.55 D	18.52	14.79	16.69	16.67 E
Means (TS)	4.68 A	3.55 C	3.95 B		22.65 A	18.18 C	20.60 B	
LSD at 5% for TS = 0.08; CS = 0.13; TS × CS = 0.22					LSD at 5% for TS = 1.56; CS = 1.23; TS × CS = NS			
HAFIZABAD								
Cotton-W	4.56 c	3.48 gh	3.97 e	4.00 C	19.44	13.51	17.93	16.96 C
Sorghum-W	3.70 f	2.70 j	3.34 h	3.25 D	16.96	11.94	14.95	14.62 D
Mungbean-W	5.52 a	4.08 de	4.64 bc	4.75 A	25.12	19.23	21.70	22.01A
Rice-W	4.81 b	3.44 gh	4.19 d	4.15 B	20.64	15.79	18.74	18.39 B
Sunflower-W	4.74 bc	3.62 fg	4.18 d	4.18 B	19.23	14.35	16.90	16.83 C
Fallow-W	3.77 f	2.55 j	3.09 i	3.14 E	14.91	10.98	12.96	12.95 E
Means (TS)	4.51 A	3.31 C	3.90 B		19.38 A	14.30 C	17.20 B	
LSD at 5% for TS = 0.09; CS = 0.11; TS × CS = 0.19					LSD at 5% for TS = 1.39; CS = 1.32; TS × CS = NS			
FAISALABAD								
Cotton-W	4.58 c	3.40 hi	3.98 fg	3.98 C	24.39	18.28	22.87	21.85 BC
Sorghum-W	4.00 e-g	2.70 k	3.48 h	3.39 D	20.45	16,83	19.87	19.05 D
Mungbean-W	5.62 a	3.83 g	4.38 cd	4.61 A	26.20	20.70	24.29	23.73 A
Rice-W	4.82 b	3.13 j	4.19 de	4.05 BC	25.70	20.59	23.75	23.35 AB
Sunflower-W	4.88 b	3.51 h	3.95 fg	4.11 B	24.19	19.17	21.79	21.71 C
Fallow-W	4.08 ef	2.68 k	3.26 ij	3.34 D	19.82	15.79	17.07	17.56 D
Means (TS)	4.66 A	3.21 C	3.87 B		23.46 A	18.56 C	21.61 B	
LSD at 5% for TS = 0.09; CS = 0.118; TS × CS = 0.21					LSD at 5% for TS = 1.78; CS = 1.51; TS × CS = NS			

Here, CT, conventional tillage; ZT, zero-tillage; MT, minimum tillage; CS, cropping systems; TS, tillage systems; W, wheat, NS, non-significant. Means followed by different letters are statistically different from each other within each location. The data of locations were analyzed separately due to differences in soil, climate and agroecology. The values presented are means of three-year data.

CT resulted in the highest number of productive tillers, number of grains per spike, 1000-grain weight, grain yield, and grain protein content at all locations, whereas the lowest values of these traits were noted under ZT. Similarly, the highest number of productive tillers, number of grains per spike, 1000-grain weight, grain yield, and grain protein content were recorded for mungbean-wheat cropping system at all locations, whereas fallow-wheat and sorghum-wheat cropping systems resulted in the lowest values of these traits. Regarding T × C interaction, mungbean-wheat cropping system with CT resulted in the highest number of productive tillers, number of grains per spike, 1000-grain weight, and grain yield, whereas fallow-wheat and sorghum-wheat cropping systems under ZT resulted in the lowest values of these traits at all locations (**Tables 4**–**6**).

The individual and interactive effects of T and C significantly altered weed density and biomass recorded at 45 and 65 days after sowing (DAS) at all locations (**Tables 7**–**9**). Wheat sown under ZT observed the highest, while wheat sown under CT recorded the lowest weed density and biomass at both sampling dates at all locations. Similarly, fallow-wheat cropping system recorded the highest weed density and biomass at 45 and 65 DAS, while sorghum-wheat cropping system recorded the lowest values for these traits, but it at par with sunflower-wheat cropping system at 45 DAS at Multan and Hafizabad locations for weed biomass. The T × C interaction revealed that fallow-wheat cropping system under ZT had the highest, whereas sorghum-wheat cropping system with CT recorded the lowest values for total weed density and biomass at both sampling dates at all locations. However, the latter was at par with sunflower-wheat cropping system under CT at 45 DAS at Hafizabad and with sorghum-wheat cropping system under MT at Faisalabad for weed biomass (**Tables 7**–**9**). The highest economic returns were noted for mungbean-wheat cropping system under conventional tillage compared to the rest of the cropping systems included in the study (data not shown).

**Table 7:** The impact of individual and interactive effects of different tillage and cropping systems on density and biomass of weed species recorded in wheat crop at Multan experimental site.

Cropping systems	CT	ZT	MT	Means (CS)	CT	ZT	MT	Means (CS)
	Multan							
	Total weed density (plants/m^2^)				Total weed biomass (g/m^2^)			
45 DAS								
Cotton-W	13.00 ij	37.00 b	19.83 f-h	23.28 C	1.07 g-i	5.37 bc	2.77 e	3.07 BC
Sorghum-W	6.50 k	21.67 e-g	12.17 j	13.44 E	0.43 i	2.10 ef	1.03 g-i	1.19 D
Mungbean-W	17.67 gh	38.00 b	25.50 de	27.06 B	1.70 fg	5.57 b	2.93 e	3.40 B
Rice-W	17.83 gh	30.00 cd	22.50 ef	23.44 C	1.23 f-i	4.47 d	2.77 e	2.82 C
Sunflower-W	12.83 ij	20.00 f-h	16.50 hi	16.44 D	0.70 hi	2.07 ef	1.63 f-h	1.47 D
Fallow-W	28.00 cd	64.67 a	38.50 b	43.72 A	2.90 e	8.90 a	4.50 cd	5.43 A
Means (TS)	15.97 C	35.22 A	22.50 B		1.34 C	4.74 A	2.60 B	
LSD at 5% for TS = 2.77; CS = 1.95; TS × CS = 4.10					LSD at 5% for TS = 0.59; CS = 0.47; TS × CS = 0.94			
65 DAS								
Cotton-W	39.00 h	85.50 c	63.50 f	62.67 C	10.13 j	26.80 c	16.17 g	17.70 C
Sorghum-W	22.50 j	64.83 ef	46.17 g	44.50 E	4.53 l	16.67 fg	10.63 j	10.61 E
Mungbean-W	49.50 g	95.50 b	71.00 d	72.00 B	11.50 ij	30.00 b	18.17 ef	19.89 B
Rice-W	48.67 g	90.83 b	70.50 d	70.00 B	7.40 k	30.47 b	18.67 e	20.83 B
Sunflower-W	33.83 i	62.17 f	50.50 g	48.83 D	13.37 h	16.57 fg	12.57 hi	12.18 D
Fallow-W	68.67 de	142.17 a	91.83 b	100.89 A	17.57 e-g	39.37 a	23.07 d	26.67 A
Means (TS)	43.69 C	90.17 A	65.58 B		10.75 C	26.64 A	16.54 B	
LSD at 5% for TS = 2.23; CS = 2.94; TS × CS = 5.13					LSD at 5% for TS = 0.93; CS = 1.05; TS × CS = 1.89			

Here, CT, conventional tillage; ZT, zero-tillage; MT, minimum tillage; CS, cropping systems; TS, tillage systems; W, wheat. Means followed by different letters are statistically different from each other within each location. The data of locations were analyzed separately due to differences in soil, climate and agroecology. The values presented are means of three-year data.

**Table 8:** The impact of individual and interactive effects of different tillage and cropping systems on density and biomass of weed species recorded in wheat crop at Hafizabad experimental site.

Cropping systems	CT	ZT	MT	Means (CS)	CT	ZT	MT	Means (CS)
	Hafizabad							
	Total weed density (plants/m^2^)				Total weed biomass (g/m^2^)			
45 DAS								
Cotton-W	18.67 ij	43.33 d	28.67 f	30.22 D	5.47 gh	12.33 d	8.47 f	8.76 C
Sorghum-W	6.67 l	24.50 gh	15.00 jk	15.39 F	1.13 k	4.30 hi	2.57 jk	2.67 D
Mungbean-W	22.00 hi	45.17 d	33.67 e	33.61 C	4.70 hi	10.67 e	8.57 f	7.98 C
Rice-W	26.33 fg	51.50 c	36.83 e	38.22 B	6.93 g	14.03 c	10.83 de	10.60 B
Sunflower-W	12.17 k	26.33 fg	17.17 j	18.56 E	2.40 jk	5.10 h	3.20 ij	3.57 D
Fallow-W	41.33 d	84.67 a	57.83 b	61.28 A	10.67 e	25.90 a	16.80 b	17.79 A
Means (TS)	21.19 C	45.92 A	31.53 B		5.22 C	12.06 A	8.41 B	
LSD at 5% for TS = 1.98; CS = 2.19; TS × CS = 3.96					LSD at 5% for TS = 0.47; CS = 0.91; TS × CS = 1.50			
65 DAS								
Cotton-W	47.50 i	84.67 d	59.33 g	64.11 C	32.60 h	56.03 c	38.57 fg	42.40 C
Sorghum-W	22.33 l	45.50 i	36.33 j	34.72 F	12.77 m	25.03 j	19.87 kl	19.22 F
Mungbean-W	45.00 i	76.00 e	59.33 g	60.11 D	30.07 hi	48.13 d	39.20 f	39.13 D
Rice-W	52.83 h	89.50 c	66.17 f	69.50 B	35.73 g	60.00 b	42.67 e	46.13 B
Sunflower-W	29.50 k	50.83 h	39.17 j	39.83 E	18.50 l	28.70 i	22.73 jk	23.31 E
Fallow-W	73.33 e	134.00 a	92.83 b	100.06 A	47.47 d	80.20 a	54.33 c	60.67 A
Means (TS)	45.08 C	80.08 A	58.94 B		29.52 C	49.68 A	36.23 B	
LSD at 5% for TS = 1.31; CS = 1.69; TS × CS = 2.96					LSD at 5% for TS = 1.87; CS = 1.75; TS × CS = 3.31			

Here, CT, conventional tillage; ZT, zero-tillage; MT, minimum tillage; CS, cropping systems; TS, tillage systems; W, wheat. Means followed by different letters are statistically different from each other within each location. The data of locations were analyzed separately due to differences in soil, climate and agroecology. The values presented are means of three-year data.

**Table 9:** The impact of individual and interactive effects of different tillage and cropping systems on density and biomass of weed species recorded in wheat crop at Faisalabad experimental site.

Cropping systems	CT	ZT	MT	Means (CS)	CT	ZT	MT	Means (CS)
	Faisalabad							
	Total weed density (plants/m^2^)				Total weed biomass (g/m^2^)			
45 DAS								
Cotton-W	13.93 gh	39.37 bc	22.60 e	25.30 B	3.71 ij	9.21 c	5.46 gh	6.13 B
Sorghum-W	5.83 j	18.53 f	10.73 hi	11.70 D	1.82 l	3.92 ij	2.40 kl	2.71 D
Mungbean-W	14.80 g	36.27 c	25.53 d	25.53 B	3.46 i-k	8.94 c	6.68 ef	6.36 B
Rice-W	16.67 fg	36.60 c	25.37 de	26.21 B	4.33 hi	8.18 cd	5.87 fg	6.13 B
Sunflower-W	9.33 ij	23.13 e	17.03 fg	16.50 C	3.20 jk	5.38 gh	3.81 ij	4.13 C
Fallow-W	28.83 d	57.50 a	42.67 b	43.00 A	7.29 de	14.86 a	10.50 b	10.88 A
Means (TS)	14.90 C	35.23 A	23.99 B		3.97 C	8.41 A	5.79 B	
LSD at 5% for TS = 0.96; CS = 2.17; TS × CS = 3.55					LSD at 5% for TS = 0.56; CS = 0.65; TS × CS = 1.16			
65 DAS								
Cotton-W	37.43 j	69.20 c	47.93 h	51.52 B	33.78 fg	48.74 c	34.73 ef	38.84 B
Sorghum-W	18.17 m	41.53 i	32.40 k	30.70 D	13.64 l	25.02 i	22.36 j	10.34 E
Mungbean-W	39.97 ij	63.100 e	50.93 g	51.33 B	31.66 g	42.15 d	31.73 g	35.84 B
Rice-W	39.00 ij	66.43 d	51.53 f	52.32 B	33.78 fg	47.96 c	36.67 e	39.47 B
Sunflower-W	26.17 l	46.97 h	38.20 j	37.11 C	19.34 k	28.45 h	24.50 ij	24.10 D
Fallow-W	55.67 g	88.17 a	73.83 b	72.56 A	42.74 d	57.03 a	51.95 b	50.57 A
Means (TS)	36.07 C	62.57 A	49.14 B		29.03 C	41.56 A	33.65 B	
LSD at 5% for TS = 1.54; CS = 1.57; TS × CS = 2.91					LSD at 5% for TS = 0.34; CS = 1.44; TS × CS = 2.30			

Here, CT, conventional tillage; ZT, zero-tillage; MT, minimum tillage; CS, cropping systems; TS, tillage systems; W, wheat. Means followed by different letters are statistically different from each other within each location. The data of locations were analyzed separately due to differences in soil, climate and agroecology. The values presented are means of three-year data.

## 4 Discussion

Soil properties (i.e., soil bulk density and soil porosity) were significantly altered by various tillage and cropping systems included in the study. As hypothesized, inclusion of leguminous crop in the cropping systems significantly improved soil health, whereas zero tillage (ZT) improved soil N availability. Overall, conventional tillage (CT) resulted in less dense and more porous soil, whereas ZT exerted opposite effect in this regard (**Table 3**). Since ZT leads to soil compaction and CT causes soil aeration, tillage methods are responsible for the observed differences in soil physical properties (Naeem et al., 2021c; Minhas et al., 2022). Yu et al. (2020) reported that soil bulk density (BD) was decreased by 5.19%, while soil porosity was improved by 5.69% under CT. The BD, soil total carbon, and soil penetration resistance have been reported to reduce with lesser tillage, whereas water infiltration rate and soil porosity were enhanced with increasing tillage intensity (Rai et al., 2018; Peixoto et al., 2020). The results of the current study revealed that fallow-wheat cropping system resulted in the highest BD and the lowest soil porosity, whereas mungbean-wheat cropping system behaved opposite in this regard (**Table 3**). The differences in the root systems and root penetration ability of different crops influence soil physical properties (Indoria et al., 2017). Mungbean is an important leguminous crop with tap root system and could reduce soil BD and improve soil porosity (Alam and Salahin, 2013). Fibrous root system of mungbean penetrates the soil and creates channels, which facilitate enhanced soil structure and porosity. Furthermore, root growth and decay results in the creation of small root cavities, which improve soil porosity. Nevertheless, mungbean emits root exudates comprising of organic acids and enzymes, that aid in the disintegration of soil aggregates and mitigate soil compaction. The improvement in soil porosity and lower BD in mungbean-wheat cropping systems are owed to the root system of mungbean crop. Recently, Shahzad et al. (2016a) and Naeem et al. (2021c) have also shown that inclusion of mungbean in different wheat- and barley-based cropping systems improved soil porosity and lowered BD. Soil porosity has a positive correlation with root growth; thus, better root growth enables plants to explore greater soil volume for nutrients and moisture absorption. The improved nutrient and water absorption leads to better plant growth and yield. The improved soil porosity in CT in the current study is owed to less compaction, breaking of hardpan and better incorporation of fertilizers.

Different tillage and cropping systems significantly affected soil available N after the harvest of wheat crop. The highest and the lowest available N was recorded for ZT and CT, respectively at all locations (**Table 4**). The ZT decreased nitrogen loss by lowering soil penetration rates and enhancing soil BD, which tends to store nutrients more securely. Hence, better nutrient contents were available in ZT than CT. Likewise, better growth of wheat crop under CT compared with ZT is linked with uptake of more nutrients, leaving small quantity of macro-and micronutrients in the soil after harvest (Issaka et al., 2019; Naeem et al., 2021c). Our findings are in agreement with Yang et al. (2015) who reported that more nutrients (N, P, and K) stayed on higher soil surface and remained intact under ZT or conservation tillage systems. Consequently, reduced nutrient loss was seen in ZT compared to CT. Moreover, mungbean-wheat cropping system observed more available soil N content, while fallow–wheat cropping system behaved oppositely (**Table 4**). Crop rotation can improve soil nutrient status by adding restorative crops like mungbean (Shahzad et al., 2016c; Naeem et al., 2021c). Cunha et al. (2011) reported that smart crop rotations may improve soil fertility status and crop nutrient absorption efficiency. Continuous use of exhaustive crops decreases soil organic carbon and nutrient availability; therefore, addition of restorative crops like mungbean improved soil nutrient status (Shahzad et al., 2016c). Moreover, legumes have the tendency to convert atmospheric N_2_ into plant available form (Ojiem et al., 2007). Therefore, these crops provide essential nutrients which are readily available to plants (Naeem et al., 2021c).

The tillage and cropping systems significantly altered weed infestation at all locations. The ZT observed heavy weed density and biomass, while CT observed lower values of these traits (**Tables 7**-**9**). Weed infestations are caused by the presence of weed seeds on topsoil, which encourages their development. Shallow or no tillage disturbs the upper soil layer without significant burial of weed seeds. It has been claimed that 60–90% of weed seeds are found on topsoil (Małecka-Jankowiak et al., 2015). The lower weed seed burial in ZT results in high volume of weed seeds in the top layer, which increases the incidence of weed infestation. As a result, weed infestation in ZT is an unavoidable fact that contradicts CT and MT (Jat et al., 2019). The CT buries weed seeds in the subsoil, which reduces weed population during the early stages of crop establishment (Chauhan et al., 2006). The CT had a great impact on weed flora and proper use of tillage can suppress weed infestation (Kadziene et al., 2020; Mehmood-Ul-Hassan et al., 2020). The differences among tillage systems regarding weed infestation are owed to seed burial and disturbance caused to the seeds in the uppermost soil layer. Minhas et al. (2022) recently revealed that CT reduced weed density and biomass compared to ZT.

Cropping system greatly affect weed infestation as sorghum-wheat cropping system recorded the lowest weed infestation, while fallow-wheat cropping system recorded the highest weed infestation and biomass at all locations (**Tables 7**-**9**). Similar trend was observed by Shahzad et al. (2016b). Addition of allelopathic crops like sorghum and sunflower in rotation discouraged the establishment of weed flora. Different allelopathic crops added to current cropping systems disrupt the cycle of weed development and reduce weed growth (Jabran and Chauhan, 2018). Crop rotation with sorghum suppress weed infestation (Farooq et al., 2020). Fallow-barley and cotton-barley cropping systems resulted in higher weed infestation in earlier studies (Naeem et al., 2021a; Naeem et al., 2021c). Employing the same farming strategy or repeatedly producing the same crop encourages the growth of more weed species compared to diversified cropping systems (Shahzad et al., 2016a).

Early crop performance is usually a significant factor determining crop yields. If a crop faces difficulties in the early stages, the yield and related traits are significantly hampered. The tillage and cropping systems had significant effect on yield-related traits of wheat crop in the current study. The CT recorded a higher number of productive tillers, number of grains per spike, 1000-grain weight, and grain yield, whereas ZT recorded lowest values of these traits (**Tables 4**-**6**). As described above, ZT had the lowest porosity and highest soil BD (**Table 3**), both of which limit root penetration. Moreover, ZT also suffered from huge weed infestation forcing crop plants to compete with weeds for light, space, water and nutrient, and deficit environment lead to poor crop performance (**Tables 4**-**6**). However, CT had the lowest BD and the highest porosity (**Table 3**), which helped in deeper root penetration resulting in higher uptake of nutrients and moisture. Furthermore, CT had less weed infestation; thus, wheat crop suffered from lesser competition ultimately resulting in improved yield and related traits (**Tables 7**-**9**). Soil is inverted in CT (Haruna and Nkongolo, 2020), which eradicates weeds (Gajri et al., 1999) and make soil well pulverized, which favor root development. Therefore, weed-crop competition for nutrient and water uptake is decreased (Chassot and Richner, 2002). Similar findings were reported by Minhas et al. (2022) that CT resulted in higher yield and related traits of wheat crop. Mungbean-wheat cropping system recorded higher values for yield and related traits, and grain protein content while sorghum-wheat and fallow-wheat cropping systems recorded the lowest values of these traits at all locations (**Tables 5**, **6**). Rahim et al. (2020) reported that inclusion of restorative crops (legumes, i.e., pulses, peas, and beans) in cropping systems can improve soil fertility. The presence of mungbean in the mungbean-wheat cropping systems of the current study improved soil physical condition (**Table 3**) and nutrient availability (**Table 4**). Mungbean-wheat cropping system disturbed weed cycle resulting in lower weed infestation and higher yield-related traits. Higher weed infestation leads to poor biomass production in fallow- and sorghum-wheat cropping systems leading to lower yield. Sorghum is a strong allelopathic crop, it releases phytotoxic chemical and phenolic compounds which suppress the growth of successive crops (Jabran, 2017b; Farooq et al., 2020). Therefore, the addition of mungbean in wheat-based cropping system enhanced soil organic matter, soil fertility (Jensen et al., 2012). Moreover, mungbean-wheat cropping system recorded higher protein contents than other cropping systems included in the current study because mungbean fixes atmospheric N and improved soil N content. Therefore, wheat crop in this cropping system recorded higher grain protein content. Nevertheless, mungbean-wheat cropping system with conventional tillage resulted in the highest economic returns. Therefore, this system could be opted to improve the system productivity. However, this inference warrants further detailed analyses as mungbean will replace rice and cotton and this could lead to reduced production of these crops. Therefore, a comprehensive system productivity and domestic demands for cotton and rice is needed to replace these crops with mungbean.

## 5 Conclusions

The results revealed that zero tillage promoted weed infestation due to minimum soil disturbance, whereas conventional tillage technique suppressed it. The fallow-wheat cropping system resulted in the highest weed infestation, while sorghum-wheat cropping system suppressed weed infestation. The mungbean-wheat cropping system improved total nitrogen content and wheat productivity. Conventional tillage resulted in higher wheat productivity due to lesser weed-crop competition. It is therefore concluded that mungbean-wheat cropping system under conventional tillage could be opted to improve wheat productivity and soil health. However, sorghum-wheat cropping system suppressed weed growth, and yield components of wheat crop. It is recommended that mungbean-wheat cropping system could be another potential cropping systems which could be opted in Pakistan. However, detailed studies relating to the possible impacts on the demand-supply relationship of cotton and rice to opt mungbean-wheat cropping system.

## Data availability statement

The raw data supporting the conclusions of this article will be made available by the authors, without undue reservation.

## Author contributions

Conceptualization, MH and MF. Methodology, MH. Software, WM. Validation, SF, HU-R, HA, and MF. Formal analysis, SF. Investigation, WM. Resources, MH. Data curation, WM. Writing—original draft preparation, WM. Writing—review and editing, HU-R, SF, MF, HA, and MH. Visualization, SF. Supervision, MH and MF. Project administration, MH. Funding acquisition, MH and HA. All authors contributed to the article and approved the submitted version.
